# Improved vessel–tissue contrast and image quality in 3D radial sampling‐based 4D‐MRI

**DOI:** 10.1002/acm2.12194

**Published:** 2017-10-04

**Authors:** Zixin Deng, Wensha Yang, Jianing Pang, Xiaoming Bi, Richard Tuli, Debiao Li, Zhaoyang Fan

**Affiliations:** ^1^ Department of Biomedical Sciences Biomedical Imaging Research Institute Cedars Sinai Medical Center Los Angeles CA USA; ^2^ Department of Bioengineering University of California Los Angeles CA USA; ^3^ Department of Radiation Oncology Cedars Sinai Medical Center Los Angeles CA USA; ^4^ MR R&D Siemens Healthineers Chicago IL USA; ^5^ MR R&D Siemens Healthineers Los Angeles CA USA; ^6^ Department of Medicine University of California Los Angeles CA USA

**Keywords:** 3D radial‐sampling, 4D‐MRI, radiotherapy planning. respiratory motion, vessel enhancement

## Abstract

**Purpose:**

In radiation treatment planning for thoracic and abdominal tumors, 4D‐MRI has shown promise in respiratory motion characterization with improved soft‐tissue contrast compared to clinical standard, 4D computed tomography (4D‐CT). This study aimed to further improve vessel–tissue contrast and overall image quality in 3D radial sampling‐based 4D‐MRI using a slab‐selective (SS) excitation approach.

**Methods:**

The technique was implemented in a 3D radial sampling with self‐gating‐based k‐space sorting sequence. The SS excitation approach was compared to a non‐selective (NS) approach in six cancer patients and two healthy volunteers at 3T. Improvements in vessel–tissue contrast ratio (CR) and vessel signal‐to‐noise ratio (SNR) were analyzed in five of the eight subjects. Image quality was visually assessed in all subjects on a 4‐point scale (0: poor; 3: excellent). Tumor (patients) and pancreas (healthy) motion trajectories were compared between the two imaging approaches.

**Results:**

Compared with NS‐4D‐MRI, SS‐4D‐MRI significantly improved the overall vessel–tissue CR (2.60 ± 3.97 vs. 1.03 ± 1.44, *P* < 0.05), SNR (63.33 ± 38.45 vs. 35.74 ± 28.59, *P* < 0.05), and image quality score (2.6 ± 0.5 vs. 1.4 ± 0.5, *P* = 0.02). Motion trajectories from the two approaches exhibited strong correlation in the superior–inferior (0.96 ± 0.06), but weaker in the anterior–posterior (0.78 ± 0.24) and medial–lateral directions (0.46 ± 0.44).

**Conclusions:**

The proposed 4D‐MRI with slab‐selectively excited 3D radial sampling allows for improved blood SNR, vessel–tissue CR, and image quality.

## 1 INTRODUCTION

Radiation therapy is one of the most common treatments for cancer.^1^, ^2^, ^3^, ^4^, ^5^, ^6^, ^7^ Patient specific treatment planning based on imaging is required for precise calculation and delivery of radiation dose to tumors while reducing dose to normal tissues.^5^, ^6^, ^7^, ^8^, ^9^ In the thoracic and abdominal regions, this is, however, complicated by respiration‐induced tumor and organ motion.^2^, ^10^, ^11^, ^12^, ^13^, ^14^ Four‐dimensional computed tomography (4D‐CT) is currently the clinical standard to quantify tumor and organ geometry and motion trajectories.^8^, ^15^, ^16^, ^17^

Despite wide clinical adaptation, 4D‐CT has a few limitations. First, intrinsically, low soft‐tissue contrast makes it difficult to visualize tumors in tissues of similar electron densities. Implanted fiducials, while useful in some cases to ameliorate the problem, are invasive and associated with imaging artifacts that may interfere with outcome assessment.^11^, ^18^ Second, 4D‐CT images, due to its two‐dimensional (2D) acquisition nature and the need for slice resorting, are prone to stitching artifacts that substantially undermine the visualization of tumors and organs.^15^, ^16^, ^19^ Third, 4D‐CT does not show adequate contrast between tumors and blood vessels. This makes it challenging to target radiation dose to the area of tumor that's in contact with the blood vessels, which is needed in, for example, pancreatic cancer patients to downstage unresectable tumors and obtain margin negative resections for significantly prolonged survival.^2^, ^9^

Alternatively, 4D magnetic resonance imaging (MRI) has been used to assess respiratory motion for radiation treatment planning.^1^, ^3^, ^5^, ^6^, ^7^ Compared to CT, MRI provides superior soft‐tissue contrast and is free of ionizing radiation. Most of previous 4D‐MRI techniques inherit the concept from 4D‐CT and are based on multiple 2D acquisitions followed by slice sorting in the image domain. The resultant images are poor in slice resolution and prone to stitching artifacts.^5^, ^6^, ^7^ Recently, continuous 3D acquisition with retrospective data sorting techniques in the k‐space domain has been proposed to provide stitching artifact‐free 4D‐MRI images with high spatial resolution.^2^, ^10^, ^12^, ^14^ However, for methods involving 3D k‐space sorting, undersampling artifacts (e.g., streaking in 3D radial acquisitions) may become evident due to drastic undersampling in patients who have a highly irregular breathing pattern, thus impairing overall image quality. In addition, to the best of our knowledge, current 4D‐MRI techniques have not looked into improvements in blood vessel delineation, which is potentially important for radiotherapy planning in pancreatic cancer patients as mentioned above. MR techniques using balanced steady‐state free precession (bSSFP) with T2‐preparation have shown to improve vessel delineation in coronary MR angiography applications.^20^, ^21^ Further, bSSFP methods have also been adopted in 4D‐MRI abdominal imaging, however, vessel–tissue contrast appears inadequate and further parameter optimization may be needed.^22^ In general, bSSFP and T2‐preparation sequences work well in lower magnetic fields, such as 1.5 T. However, at 3.0 T, these techniques may be susceptible to field inhomogeneity and more prone to image artifacts.

In this study, we aimed to develop an improved vessel‐enhanced method at 3.0 T using a slab‐selective 3D radial sampling‐based gradient recalled echo (GRE) acquisition approach. In addition, improvement in image quality was also explored for the proposed technique. A pilot study including both healthy subjects and patients was performed to demonstrate these technical improvements.

## 2 METHODS AND MATERIALS

### 2.A Sequence development

In 3D radial sampling‐based methods, non‐selective (NS) excitation with hard radio‐frequency (RF) pulses is typically used to excite a volume considerably larger than the prescribed field of view (FOV). As a result, blood spins will experience a large amount of RF pulses prior to entering the FOV and thus exhibit a substantially decayed signal level and reduced contrast to stationary tissue spins.

**Figure 1:**
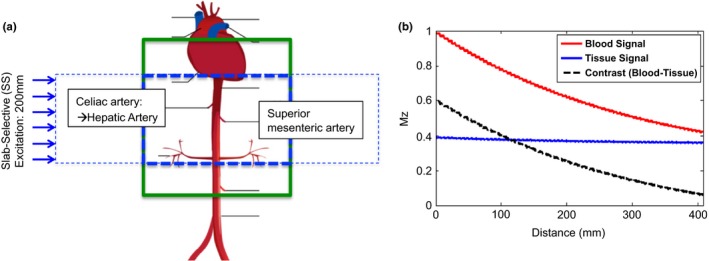
(a) Schematic of non‐selective (NS) excitation (applied on a volume considerably larger than the prescribed field‐of‐view shown as the green‐solid box) and slab‐selective (SS) excitation (applied on the blue‐dash box). (b) The longitudinal magnetization in the blood and tissue vs. the blood spins’ traveling distance (TD). The blood longitudinal magnetization decreases with TD, whereas the tissue longitudinal magnetization remains in a steady state due to repetitive RF excitations. Therefore, vessel–tissue contrast also decreases with TD. Blue‐dash box represents the SS excitation scenario where sufficient contrast still remains throughout the imaging volume. Asc. A: Ascending aorta; TA: Thoracic aorta; HA: Hepatic artery; SA: Splenic artery; AA: Abdominal aorta; SMA: Superior mesenteric artery; IMA: Inferior mesenteric artery; FOV: field‐of‐view.

Slab‐selective (SS) excitation, in contrast, would ensure fresh blood spins entering the excited volume, thus improving the contrast between blood vessels and stationary tissues. The schematic diagram of NS and SS excitation approaches and simulations of blood and tissue signal and their contrast vs. the blood spins’ traveling distance are shown in Fig. 1. In simulations, a general GRE signal equation was used with T1 values of 1500 and 725 ms for the blood and tissue, respectively. The velocity of the blood was assumed as 1 m/s to reflect the scenario in the abdominal aorta. As shown in Fig. 1(b), the blood signal (equivalently represented by the longitudinal magnetization) gradually decreases with the blood spins’ traveling distance, whereas the stationary tissue signal remains in a steady state due to repetitive RF excitations; vessel–tissue contrast is well preserved with the in‐flow effect by using the SS excitation approach (the blue‐dash box). Of note, slower blood flow and tortuousness of the smaller blood vessels may experience more RF excitations, which may lead to a faster signal decay and reduced vessel–tissue contrast than shown in simulations herein.

In addition to potential improvements in vessel–tissue contrast, the SS approach may also help suppress the signals form peripheral structures in the superior–inferior direction. These signals would otherwise contribute to streaking artifacts in the FOV when drastic k‐space undersampling occurs.

Hence, an SS‐excited 3D radial sampling‐based technique was developed in order to achieve the improvements above. The MRI sequence was implemented on the basis of a 4D‐MRI framework–3D radial sampling with self‐gating‐based k‐space sorting (SG‐KS).^2^, ^23^

### 2.B Experiments

To evaluate the performance of the SS‐excited 3D radial sampling‐based 4D‐MRI technique (SS‐4D‐MRI), six patients (mean age: 63.5 ± 17.2 yr; all males; 1 lung, 1 liver, 1 esophagus, and 3 pancreatic) and two healthy subjects (mean age: 36.5 ± 0.7 yr; 1 female) were prospectively recruited with an institutional review board approval and informed consent. Two lesions in the lung patient, three lesions in the liver patient, and a single lesion in all other patients (esophagus and pancreatic) were present.

All imaging studies were performed on a hybrid PET‐MRI system with a 3.0‐Tesla magnetic strength (Biograph mMR, Siemens Healthcare, Germany) using a body matrix coil and spine coils. The advantages of the SS excitation approach were investigated through a comparison study involving SS‐4D‐MRI and NS‐4D‐MRI scans. For a fair comparison, the two scans employed the same SG‐KS 4D‐MRI framework.^2^, ^13^ Specifically, a GRE sequence with 3D radial k‐space sampling was used for data acquisition. Self‐gating was used for respiratory motion detection and 2D golden‐means k‐space trajectory was used to enable arbitrary k‐space sorting. Acquired k‐space data were sorted into ten respiratory bins and then underwent conjugate gradient sensitivity encoding reconstruction combined with iterative motion correction and averaging.^8^, ^17^ SS excitation was prescribed in an axial orientation with a slab thickness of 200 mm (sufficient to cover variously sized tumors in a single organ). The shared imaging protocol for SS‐4D‐MRI and NS‐4D‐MRI experiments were as follows: FOV = (400 mm)^3^; prescribed spatial resolution = (1.56 mm)^3^; flip angle = 12^o^; repetition time/echo time = 5.5/2.68 ms; readout bandwidth = 429 Hz/pixel; scan time = 7:23 min with 80,020 projections; To detect respiratory motion, SG lines were inserted at every 18 imaging lines or at intervals of approximately 110 ms. All imaging lines were then sorted where ten temporally binned respiratory phases were reconstructed for image analysis.

### 2.C Image Analysis

#### 2.C.1 Evaluation of vessel‐tissue contrast

To evaluate the effect of SS excitation on vessel delineation, vessel–tissue contrast was quantitatively compared between (1) SS‐4D‐MRI and NS‐4D‐MRI, (2) different vessel locations through the imaging volume, (3) different vessel size (aorta, superior mesenteric artery [SMA], inferior vena cava [IVC], and superior mesenteric vein [SMV]), and (4) artery and vein in five (three pancreatic cancer patients and two healthy subjects) of the eight subjects. Signal intensities are measured in a region of interest (ROI) of a specified blood vessel such as the aorta, IVC, SMA, or SMV and of its nearby tissue such as the pancreas, tumor, or other nearby organs. Contrast ratio (CR) is calculated as the signal difference between blood vessel and its nearby tissue divided by the nearby tissue signal. Signal‐to‐noise ratio (SNR) is calculated as the ratio of the blood vessel signal‐to‐noise (standard deviation of ROI located in a fixed air space). For comparison (1), (2), and (4), measurements were performed in the aorta and IVC at slices located 50, 100, and 150 mm into the 200 mm SS excitation slab. For comparison (3), measurements were performed in a slice within the central pancreas region (near the center of FOV) where all four vessels (aorta, SMA, IVC, and SMV) were visible in one single plane. In addition, overall SNR of both SS‐ and NS‐4D‐MRI methods were compared in the large vessels. All measurements were performed in the axial images.

#### 2.C.2 Evaluation of streaking artifacts

Overall image quality, primarily the severity of streaking artifacts, was qualitatively compared between the SS‐4D‐MRI and NS‐4D‐MRI approaches in all subjects. All 4D image datasets were randomized and blindly reviewed in consensus by two experienced reviewers (one medical physicist with 10 yr of experience in radiation therapy planning and one MRI physicist with 6 yr of experience in abdominal imaging) using the OsiriX Imaging Software (OsiriX; Pixmeo SARL, Switzerland). A 4‐point scale was used: 0, poor (severe streaking artifacts or signal loss and difficult visualization of relevant anatomic structures); 1, fair (visible streaking artifacts or signal loss but acceptable visualization of relevant anatomic structures); 2: good (minor streaking artifacts or signal loss and good visualization of relevant anatomic structures; 3: excellent (no visible streaking artifacts or signal loss and excellent visualization of relevant anatomic structures).

#### 2.C.3 Evaluation of motion trajectory

To ensure that the motion information is maintained when using SS‐4D‐MRI, motion trajectories of the tumors (pancreas, liver, lung, or esophagus) in all patients and the pancreas in healthy subjects were obtained from the SS‐4D‐MRI and NS‐4D‐MRI images, respectively. Specifically, tumor and pancreas ROIs were contoured on the end of exhalation dataset. Three‐dimensional deformable image registration based on the B‐spline algorithm was then performed across all respiratory bins using Velocity^TM^ (Varian Medical Systems, Palo Alto, CA, USA). The coordinates at the geometric center of each contour were extracted from each respiratory bin and were used to determine the motion trajectories.

### 2.D Statistical analysis

SPSS v.16.0 (SPSS Inc., Chicago, IL, USA) was used for statistical analysis. Wilcoxon signed rank test was used to determine the differences in blood vessel SNR, vessel–tissue CR, and image quality score between the SS‐4D‐MRI and NS‐4D‐MRI approaches. Intraclass correlation was used to determine the agreement in the motion trajectory between the two approaches. In all tests, statistical significance was defined at *P <* 0.05 and data were presented as means ± standard deviations.

## 3 RESULTS

**Figure 2:**
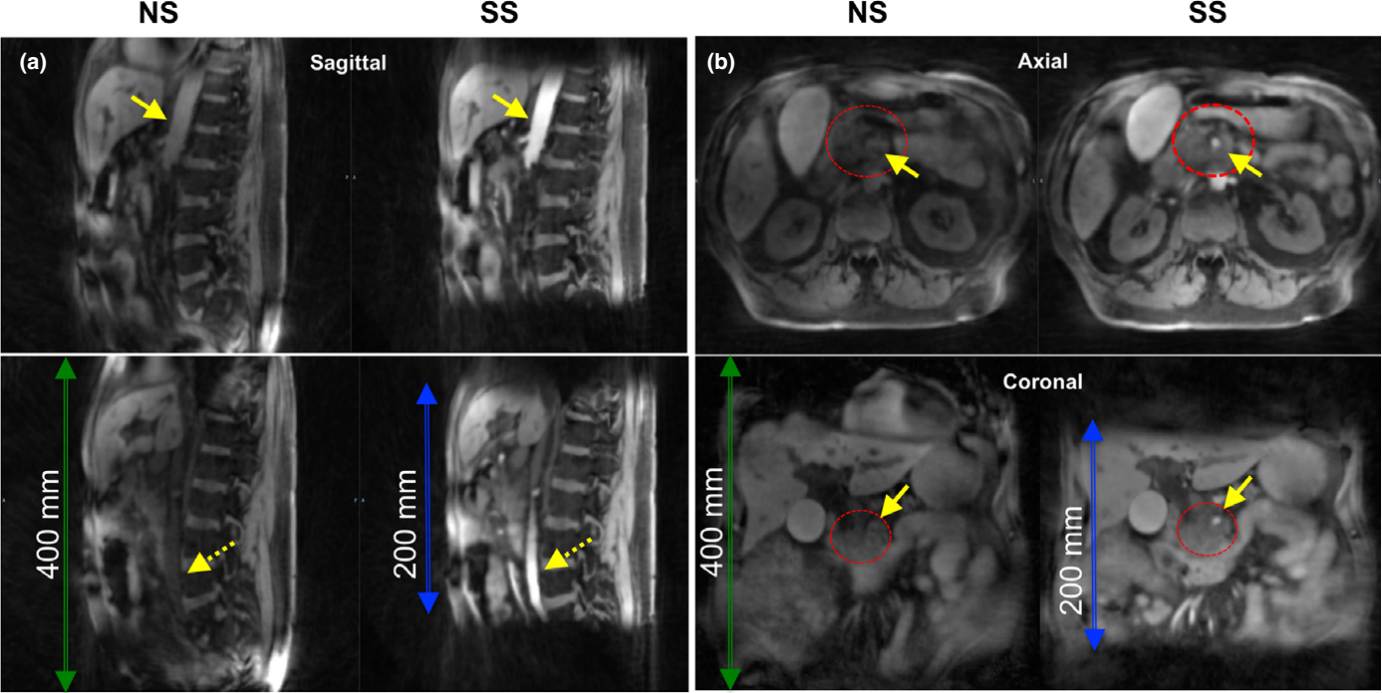
(a) Large blood vessel (aorta (solid arrow) and inferior vena cava (dashed arrow)) to tissue contrast of non‐selective (NS)‐4D‐MRI vs. slab‐selective (SS)‐4D‐MRI in a sagittal view. (b) small blood vessel (superior mesenteric artery, SMA, solid arrows) to tissue contrast of NS‐4D‐MRI vs. SS‐4D‐MRI in an axial and coronal view. The dashed circle denotes the pancreatic tumor location for this particular patient.

All subjects successfully underwent SS‐4D‐MRI and NS‐4D‐MRI scans. Figure 2 shows the visual comparisons of vessel–tissue contrast between SS‐4D‐MRI and NS‐4D‐MRI approaches in both large and small vessels of a pancreatic cancer patient. Large (aorta and IVC) and small (SMA) blood vessels are well delineated in the SS‐4D‐MRI approach compared to the NS‐4D‐MRI approach.

**Figure 3:**
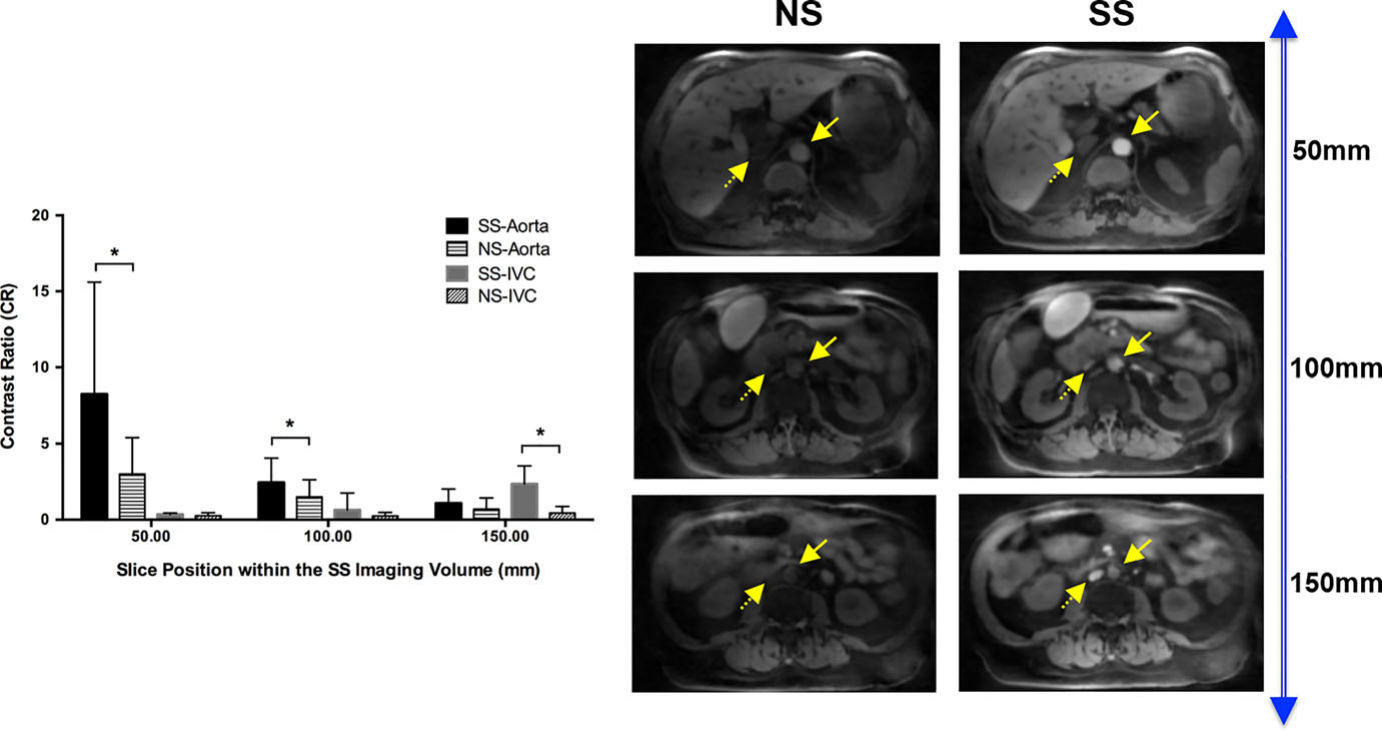
Vessel–tissue CR at 50, 100, and 150 mm of a total 200 mm SS excitation imaging volume in the aorta and IVC for SS‐4D‐MRI and NS‐4D‐MRI methods. Solid arrows points at the aorta; dashed arrows point at the IVC; * represents statistical significance (*P* < 0.05); IVC = inferior vena cava; SS = slab‐selective; NS = non‐selective.

**Figure 4:**
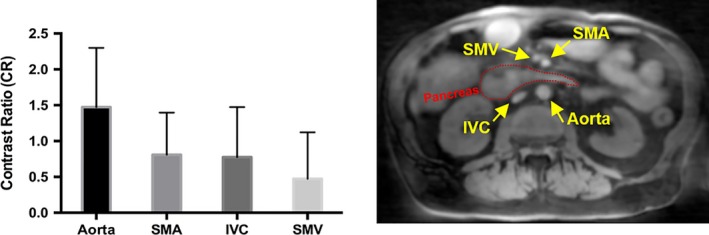
Comparison between different vessels (aorta, SMA, IVC, and SMV) in one plane near the pancreas or tumorous region. Example image is labeled with its representative vessels. SMA = superior mesenteric artery; SMV = superior mesenteric vein; IVC = inferior vena cava.

Overall, significantly (*P <* 0.05) higher SNR was observed in the SS‐4D‐MRI (63.33 ± 38.45) method compared to NS‐4D‐MRI (35.74 ± 28.59). Figures 3 and 4 shows the quantitative effects of SS excitation on vessel delineation. Figure 3 shows vessel–tissue CR at the location of 50, 100, and 150 mm in the aorta and IVC for SS‐4D‐MRI and NS‐4D‐MRI methods. Averagely, significantly (*P <* 0.05) higher CR was observed in the SS‐4D‐MRI (2.60 ± 3.97) method compared to NS‐4D‐MRI (1.03 ± 1.44) method. In the aorta, a gradual signal decay was observed when moving from the SS imaging volume location of 50 mm to the location of 150 mm in the SS‐4D‐MRI method (87.7%), and similar trend was also observed in the NS‐4D‐MRI method (76.8%). When comparing between the SS‐ and NS‐4D‐MRI methods in the aorta, significantly higher vessel–tissue CR was found in SS‐4D‐MRI images at the location of 50 mm (8.47 ± 7.22 vs. 3.01 ± 2.39; *P =* 0.043) and 100 mm (2.46 ± 1.60 vs. 1.51 ± 1.12; *P =* 0.043), where at the location of 150 mm two techniques were not significantly different (1.04 ± 0.96 vs. 0.70 ± 0.74; *P =* 0.225). In the IVC, as blood flow is from inferior to superior, a gradual signal decay was also observed from the SS imaging volume location of 150 mm to the location of 50 mm in the SS‐ (83.9%) and NS‐ (38.2%) 4D‐MRI methods. When comparing between the two methods in the IVC, significantly higher vessel–tissue CR was found in SS‐4D‐MRI images at the location of 150 mm (2.53 ± 0.96 vs. 0.44 ± 0.74; *P* = 0.043) and not statistically significant in other locations. Figure 4 shows the comparison between different vessels (aorta, SMA, IVC, and SMV) in one plane near the central pancreas region. When comparing between large (aorta/IVC) to small (SMA/SMV) vessels, overall vessel–tissue CR was reduced by 45.2% in arteries and 38.8% in veins. No statistical significant differences were found when comparing between large (aorta/IVC) and small (SMA/SMV) vessels.

**Figure 5:**
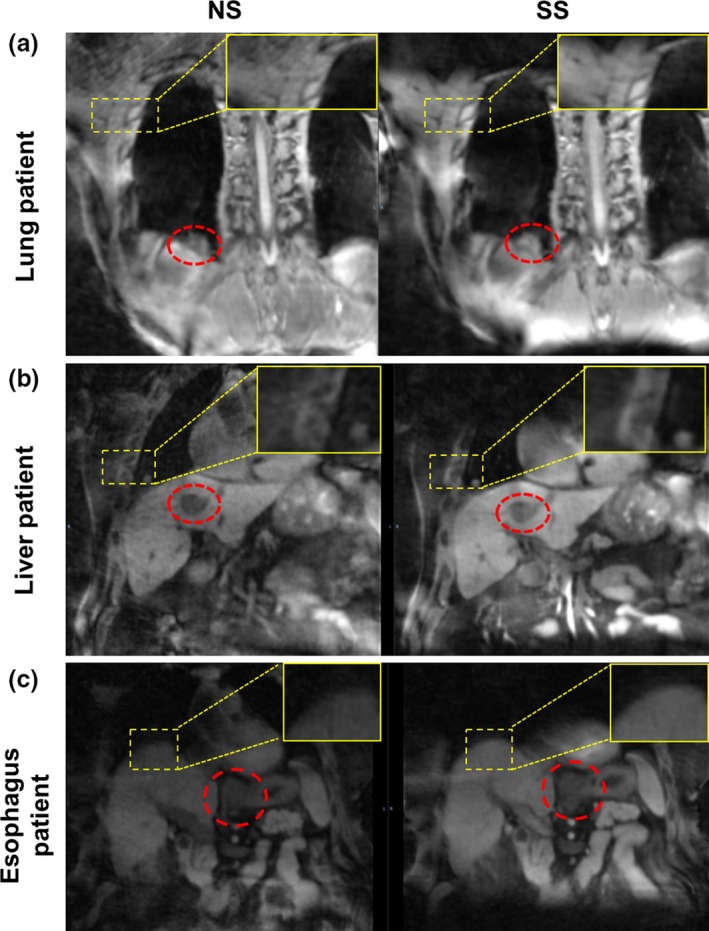
Image quality comparison between non‐selective (NS)‐4D‐MRI and slab‐selective (SS)‐4D‐MRI approaches in a (a) non‐small cell lung cancer patient with image score of 2 for SS and 1 for NS, (b) liver cancer patient with image score of 2 for SS and 1 for NS, and (c) esophagus cancer patient with image score of 3 for SS and 1 for NS. Red circles denote the tumor region in that particular patient.

**Figure 6:**
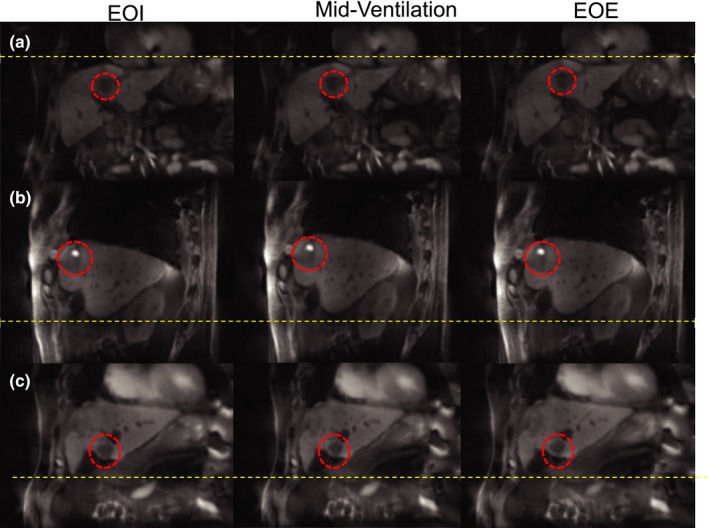
Example SS‐4D‐MRI images of respiratory phases at end of inspiration (EOI), mid‐ventilation, and end of expiration (EOE) in a liver patient with coronal (a,c) and sagittal (b) views of different tumor lesions. The red circles represent tumor location.

Figure 5 shows the image quality comparison between SS‐4D‐MRI and NS‐4D‐MRI approaches in three different patients (lung, liver, and esophagus). Less streaking artifacts and associated blurring or signal dropout were observed in the SS‐4D‐MRI images. Figure 6 shows SS‐4D‐MRI images at end of inspiration, mid‐ventilation, and end of expiration respiratory phases reformatted from the 4D‐MRI image series in a liver cancer patient. Different tumor lesions are well delineated in each respiratory phase.

Table 1 summarizes the image quality scores and correlation coefficient of respiratory motion trajectory agreement between SS‐4D‐MRI and NS‐4D‐MRI in all subjects. Significantly, fewer streaking artifacts from k‐space undersampling were observed in SS‐4D‐MRI images compared to NS‐4D‐MRI images (image quality score: 2.6 ± 0.5 vs. 1.4 ± 0.5, *P =* 0.02). Strong correlation was observed in the superior–inferior (SI) direction (0.96 ± 0.06) and weaker was seen in the anterior–posterior (AP) (0.78 ± 0.24) and medial–lateral (ML) (0.46 ± 0.44) directions among all subjects. Lower correlation in the AP and ML directions may be due to the small range of motion relative to the image spatial resolution.

**Table 1:** Patient image quality score and correlation coefficient of respiratory motion trajectory summary

No.	Age (y)	Sex	Tumor location	SS/NS	Motion trajectory		
				Image score	Correlation coefficient		
					SI	AP	ML
1	50	M	Esophagus	3/1	1.00	0.95	0.27
2	73	M	Lung	2/1	0.98	0.53	0.85
3	75	M	Liver	2/1	0.92	0.83	0.11
4	69	M	Pancreas	3/1	0.97	0.70	0.63
5	79	M	Pancreas	2/2	0.96	0.95	‐0.26
6	35	M	Pancreas	3/1	0.96	0.99	0.88
7	37	F	X	3/2	0.99	0.43	1.00
8	36	M	X	3/2	1.00	0.99	0.49
Mean (STD)	56.8 (19.2)			2.6/1.4 (0.5)/(0.5)	0.96 (0.06)	0.78 (0.24)	0.46 (0.44)

SS, slab‐selective; NS, non‐selective; SI, superior–inferior; AP, anterior–posterior; ML, medial–lateral; STD, standard deviation.

## 4 DISCUSSION

This study presents a new 4D‐MRI technique with improved vessel–tissue contrast and reduced streaking artifacts. Compared to the typically used NS excitation approach in 3D radial sampling, the proposed SS excitation approach markedly improved blood vessel SNR, vessel–tissue CR, and reduced image artifact. This was achieved without compromising SG‐KS 4D‐MRI's ability to quantify tumor and normal organ motion trajectories at both thoracic and abdominal sites.

The blood vessel highlighting technique is particularly useful for pancreatic cancer patients. Pancreatic ductal adenocarcinoma is an aggressive disease with high mortality rates. Complete surgical resection is the most effective treatment, however, the primary tumor often surrounds or encases the vasculature, thereby making the tumor unresectable in most cases.^11^, ^18^ Radiation therapy with simultaneous integrated boost has been proposed to improve tumor resection rate by delivering a greater sterilizing dose to tissues surrounding the vessels but its success clearly depends on how accurate these vessels can be localized and motion characterized.^15^, ^16^, ^19^ Due to the low contrast of vessels in both noncontrast 4D‐MRI and 4D‐CT, this has been a major roadblock for pancreas radiotherapy that requires repeated imaging.

The SS‐4D‐MRI approach exploits the effect of flow‐related enhancement. As fresh blood first enters the imaging volume of interest and experiences fewer RF pulses than stationary tissues, blood signal is markedly higher than that of tissue, creating appreciable vessel–tissue contrast. This phenomenon applies to both arteries and veins as shown in Fig. 2(a). In this study, a 200‐mm slab thickness was used as this imaging volume appeared sufficient to cover variously sized tumors in a single organ. Adequate vessel–tissue contrast was observed in the tumor involved vessels, especially near the aorta. Images from SS‐4D‐MRI showed overall higher vessel–tissue contrast compared to images from NS‐4D‐MRI. The SS‐4D‐MRI technique shows the potential of providing such information without the need for repeated administration of intravenous contrast for fractionated radiotherapy.

As an additional benefit of SS excitation, image quality was also improved with evidently reduced streaking artifacts, which is otherwise likely present with drastic k‐space undersampling in conventional 3D radial sampling and k‐space sorting‐based 4D‐MRI. By exciting a smaller imaging volume with the SS‐4D‐MRI approach, the excess signal from the superiorly and inferiorly peripheral structures has less contribution to the reconstructed images, thus, reducing the amount of streaking artifacts normally seen in the NS excitation‐based methods. Hence, SS‐4D‐MRI could potentially afford higher undersampling ratio (shown in the supplemental file) for patients with irregular breathing patterns, who may require high data rejection rates, or permit shorter imaging time for patients with stable breathing. However, severe breathing abnormality was not observed in the small subject group, and thus the potential improvement with the proposed method was not systematically investigated.

The technique has several limitations which could be improved. The proposed SS‐4D‐MRI approach is mainly dependent on the in‐flow effect for vessel enhancement, which leads to a gradual decrease in blood signal as flowing spins traverse the slab. In addition, as blood flow velocity is dependent on vessel size and location, vessel–tissue contrast may differ from large to small vessels within a subject. Furthermore, blood flow velocity may vary from subject to subject, resulting in variable vessel–tissue contrast from subject to subject. These issues could potentially be alleviated by using optimal placement of the selective slab or flexible slab thickness. One can place the selective slab so that the blood vessel of interest is in close proximity to its upstream edge of the slab, thus maximizing the in‐flow effect. This is particularly beneficial for slow‐flow small vessels. Depending on the imaging volume of interest, flexible slab thickness could also help to achieve desired vessel–tissue contrast.

This study was performed in a small cohort of heterogeneous subjects to show the feasibility of enhancing vessel–tissue contrast. A study with a larger number of patients with blood vessel‐involved pancreatic cancer is needed to clinically evaluate the efficacy of the vessel contrast‐boosting approach in treatment outcome for this particular patient population.

## 5 CONCLUSIONS

A SS‐excited radial sampling‐based 4D‐MRI technique was developed and tested in healthy volunteers and cancerous patients. The technique significantly improves vessel–tissue contrast and image quality, resulting in a set of respiratory‐resolved 3D volumetric MRI images with high isotropic resolution and superior soft‐tissue and vessel–tissue contrast.

## CONFLICT OF INTEREST

The authors have no relevant conflicts of interest to disclose.

## Supporting information

Data S1: SS‐4D‐MRI versus NS‐4D‐MRI approach at different undersampling ratios.
