# Supplementary material for: Improved vessel–tissue contrast and image quality in 3D radial sampling‐based 4D‐MRI
Source: J Appl Clin Med Phys. 2017 Oct 4;18(6):250–7. doi: 10.1002/acm2.12194 (PMC5689937; doi:10.1002/acm2.12194)
Supplement: Supplementary file 1 — Data S1: SS‐4D‐MRI versus NS‐4D‐MRI approach at different undersampling ratios. [file ACM2-18-250-s001.docx]

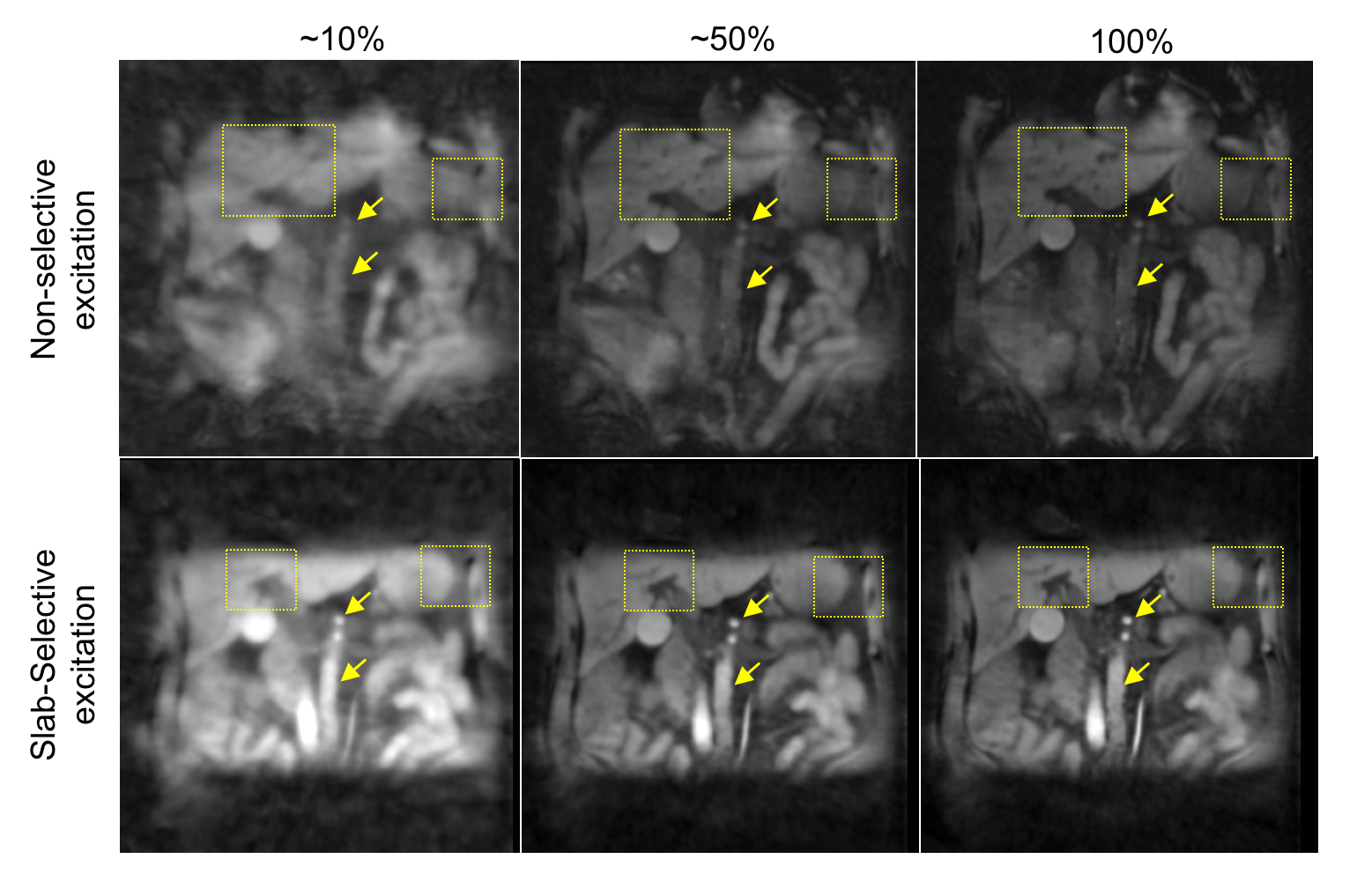


The authors investigated the differences between the SS-4D-MRI and NS-4D-MRI approach under different undersampling ratios. Image sets were reconstructed with approximately 10% and 50% of the total data. As shown in the figure above, when using the SS-4D-MRI approach, near identical image quality (dashed boxes) and preserved vessel-tissue contrast (arrows) were observed even after 50% data reduction, showing the robustness of the technique. In contrast, the NS-4D-MRI approach showed slight streaking artifacts and image blurring (dashed boxes) after 50% data reduction. In images with approximately 10% of the total data, severe image blurring and appreciable streaking artifacts were observed in the NS-4D-MRI images making it hard to differentiate certain structures, whereas good vessel-tissue contrast and delineation of overall organ structures were still preserved in the SS-4D-MRI images. This figure demonstrates the robustness of the SS-4D-MRI approach to undersampling.

Note: As SS-4D-MRI and NS-4D-MRI are two separate scans, the authors made their best effect to match the imaging slices between NS-4D-MRI and SS-4D-MRI in the figures.
